# Psychotic-like experiences in the lonely predict conspiratorial beliefs and are associated with the diet during COVID-19

**DOI:** 10.3389/fnut.2022.1006043

**Published:** 2022-10-28

**Authors:** Damiano Terenzi, Anne-Katrin Muth, Annabel Losecaat Vermeer, Soyoung Q. Park

**Affiliations:** ^1^Department of Decision Neuroscience and Nutrition, German Institute of Human Nutrition (DIfE), Potsdam-Rehbrücke, Germany; ^2^Neuroscience Research Center, Charité - Universitätsmedizin Berlin, Corporate Member of Freie Universität Berlin, Humboldt-Universität zu Berlin and Berlin Institute of Health, Berlin, Germany; ^3^Deutsches Zentrum für Diabetes, Neuherberg, Germany

**Keywords:** conspiratorial beliefs, psychotic-like experiences, loneliness, diet, COVID-19

## Abstract

The COVID-19 pandemic has increased the occurrence of conspiracy theories. It has been suggested that a greater endorsement of these theories may be associated with psychotic-like experiences (PLEs), as well as with social isolation. In this preregistered study, we investigated whether both PLEs and measures of social isolation (e.g., loneliness) can predict conspiratorial beliefs and, if so, which of these variables can mediate the association with conspiratorial beliefs. Furthermore, based on previous studies on schizophrenia, we explored whether the diet is associated with PLEs and conspiratorial beliefs. Participants (*N* = 142) completed online questionnaires measuring PLEs, social isolation, mental well-being, and conspiratorial beliefs. They also submitted their daily food intake for a week using a smartphone app. We found that loneliness predicted the endorsement of conspiracy theories during the COVID-19 lockdown. Strikingly, the proneness to experience subclinical psychotic symptoms played an underlying mediating role. In addition, these subclinical symptoms were associated with lower fruit, carbohydrate, and iron intakes, as well as with higher fat intake. Our results add insights into how conspiratorial beliefs can affect individuals’ mental health and relationships. Moreover, these results open the avenue for potential novel intervention strategies to optimize food intake in individuals with PLEs.

## Introduction

The COVID-19 pandemic has affected almost all aspects of human societal daily life. A typical psychological reaction to a highly uncertain situation such as a pandemic is the increased occurrence of conspiracy theories (1, 2). These theories are alternative explanations of important events as the results of malevolent actions (or patterns of secret causal connections) involving small powerful groups, when other explanations are more plausible (3).

During the COVID-19 pandemic, the overload of COVID-19 related information, the lack of knowledge about the disease, and the more general climate of uncertainty have given rise to conspiracy theories (4). Believing in such theories may have several detrimental effects. For example, conspiracy theories linking the 5G cellular network with COVID-19 have led to episodes of violence against telecom workers in the U.K. (5). Further, conspiratorial beliefs increase vaccine refusals (6) and decrease compliance with preventive measures such as social distancing (5). Given their impact on individuals’ health and safety, it is essential to identify the factors associated with beliefs in conspiracy theories.

In view of these detrimental consequences, recent studies have found that a greater endorsement of conspiracy theories is associated with psychotic-like experiences (PLEs) (7, 8). More in detail, PLEs refer to subclinical psychotic events (e.g., subthreshold forms of paranoid delusions) experienced by healthy individuals in the general population in the absence of a clear psychotic disorder (9, 10). Studies on the general adult population have found that approximately 8% of individuals who reported PLEs will become clinically psychotic after 2 years (11), suggesting that PLEs may represent a risk factor for developing psychotic disorders.

Interestingly, paranoia and conspiracy theories seem to have in common an intuitive thinking style and the so called “jumping to conclusion” bias, which is the tendency to make quick decisions based mostly on a few pieces of evidence (8). Thus, PLEs may be an indicator of the latent liability for conspiratorial beliefs. Moreover, several studies have found links between psychotic disorders and environmental factors such as a poorer diet quality (e.g., lower intake of fruit and vegetable). Some evidence has particularly identified iron deficiency as one of the most important dietary risk factor for psychosis (12, 13). Indeed, iron deficiency due to a reduced iron intake may alter prefrontal dopaminergic transmission in the brain leading to negative symptoms in schizophrenia (12, 14). Since food is a modifiable risk factor, it may be possible that nutritional interventions may prevent the occurrence also of PLEs and consequently reduce the susceptibility to believe in conspiratorial theories. However, empirical evidence is lacking.

Besides PLEs, another factor that has been linked to conspiratorial beliefs is social isolation (7). In particular, contention measures during the COVID-19 pandemic such as lockdowns and social distancing have influenced the quantity and quality of social interactions and enormously increased feelings of loneliness (15). Loneliness refers to perceived social isolation and is associated with poorer mental health including stress (16) and PLEs (17). Interestingly, some studies showed that loneliness could continue even when the lockdowns ended (15) and that the development of mental health problems can further strengthen the magnitude of loneliness (18). Previous research on ostracism, a form of social exclusion, has suggested that one of its most important consequences is indeed conspiratorial thinking (19, 20). Thus, the social exclusion experienced during the COVID-19 lockdown could have led people to endorse conspiracy theories.

Based on the above-mentioned studies, conspiratorial beliefs may be associated with several interconnected factors including PLEs, social isolation, and a more general reduced mental well-being. However, not all individuals experiencing social isolation or having PLEs may believe in conspiracy theories. Yet, it is unknown which factors may interact with each other and play a role in making people more susceptible to believing in conspiracy theories.

**FIGURE 1:**
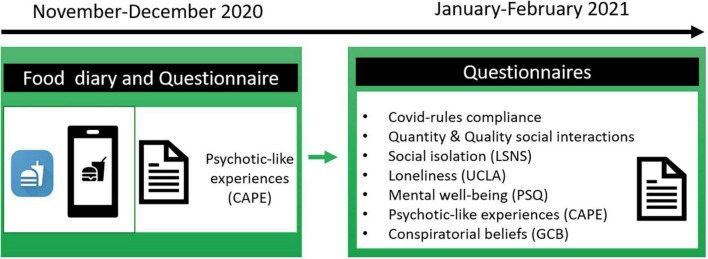
Study outline. Participants first answered a questionnaire assessing psychotic-like experiences (PLEs) and completed a daily food diary for 7 days *via* a smartphone app. Subsequently, they completed questionnaires measuring social isolation, conspiratorial beliefs, mental well-being, PLEs, demographics among others.

In this preregistered study, we first investigated whether conspiratorial beliefs during a global health crisis, such as the COVID-19 pandemic, are associated with the proneness to present PLEs. We hypothesized that during times encompassing high loneliness and uncertainty (21), individuals who report PLEs are more susceptible to believe in conspiracy theories. Second, we examined how other pandemic-related factors such as social isolation and mental well-being may relate to conspiracy theories beliefs and PLEs. We hypothesized that all these variables may be associated with each other. If so, we will then perform separate mediation analyses to determine the possible mechanisms by which PLEs, social isolation or mental well-being may relate to the endorsement of conspiracy theories. Lastly, based on previous research on schizophrenia we will explore possible associations between diet, PLEs and conspiratorial beliefs. We postulate that people reporting low average iron intake are also more prone to show PLEs as well as to believe in conspiracy theories. To test these hypotheses, participants completed questionnaires assessing PLEs, social isolation (loneliness, social support, quantity, and quality of social interactions), mental health (e.g., stress), and conspiratorial beliefs. They also submitted their daily food intake for 7 days using a smartphone app (see Figure 1 and section “Materials and methods”).

## Materials and methods

### Procedure

After providing instructions, participants were invited to complete a battery of online questionnaires assessing PLEs, social isolation (loneliness, social support, quantity, and quality of social interactions), mental health (e.g., stress), and conspiratorial beliefs see Figure 1. Written informed consent was obtained from each participant. Data were collected between 29 January and 8 February 2021) at which time there was a strict lockdown in Germany and Austria.

We also previously collected (10 November–23 December 2020) from the same participants, as a part of a larger study (preregistered under),^1^ a daily diary of their food consumption. In particular, participants were asked to install a food-diary app provided by us on their smartphone, and submit their daily intake of food items and beverages in the app, from which we extracted the total calories, and the macro- and micronutrients per meal per day (22) (see Figure 1).

### Participants

An initial sample of 147 participants took part in the study. Participants recruitment was completely online *via* Prolific,^2^ including invitations and data collection. Inclusion criteria were: (1) residing in Germany or Austria, (2) being fluent in German, and (3) no personal history of psychiatric illness.

Five participants were excluded from the analyses, as they were not residing in Germany or Austria. Thus, the final sample included 142 participants (see Table 1 for demographics). All subjects were paid £3.50 for their participation. The Humbold Ethics Committee approved the study, which was conducted in accordance with the Declaration of Helsinki.

**TABLE 1:** Sociodemographic variables and questionnaires data.

	*N* = 142
**Demographics**	
Age	29.04 (8.74); 18.00–68.00
Gender (female)	57
Education (years)	14.79 (2.14); 11.00–17.00
BMI	23.86 (4.35); 14.69–41.97
**Living situation**	
COVID-rules compliance (0–100)	82.47 (22.07); 0.00–100.00
**Psychotic-like Experiences**	
CAPE-positive	2.60 (0.53); 2.05–5.65
CAPE-negative	3.93 (0.94); 2.14–6.64
CAPE-depressive	3.87 (0.91); 2.00–6.25
CAPE-total score	10.40 (2.00); 6.64–17.70
**Mental well-being**	
PSQ	42.60 (1.48); 3.33–90.00
**Social isolation**	
Quantity social interactions (0–100)	46.23 (28.33); 0.00–100.00
Quality social interactions (0–100)	65.51 (25.37); 0.00–100.00
LSNS	13.77 (4.14); 3.00–24.00
UCLA	44.98 (14.44); 21.00–88.00
**Conspiracy beliefs**	
GCB	26.62 (10.78); 15.00–72.00

Mean (SD); range. CAPE, community assessment of psychic experiences; PSQ, perceived stress questionnaire; LSNS, Lubben Social Network Scale; UCLA, University of California Los Angeles loneliness scale; GCB, generic conspiracist beliefs scale.

### Questionnaires

#### Psychotic-like experiences

The Community Assessment of Psychic Experience (CAPE) is a 42-item questionnaire that measures self-reported subclinical psychotic symptoms in the general population based on three dimensions: positive symptoms, negative symptoms, and depression (23, 24). Several studies have shown that the CAPE can be a screening tool to identify people who might be at risk for psychosis (9, 10, 25).

#### Conspiratorial beliefs

The Generic Conspiracist Beliefs Scale (GCB) (26) includes 15 questions relating to different conspiracy theories and asks respondent how much they agree with each given statement on a five-point scale. This scale has a total score ranging from 15 to 75, with higher scores reflecting higher levels of conspiracy beliefs. The GCB is one of the most largely used measure of beliefs in conspiracy theories (27) and comprises distinct but related factors such as Government Malfeasance, Extraterrestrial Cover-up, Malevolent Global Conspiracies, Personal Wellbeing, and Control of Information (27, 28).

#### Mental well-being

The Perceived Stress Questionnaire (PSQ-20) is a psychological instrument measuring subjective experiences of perceived stress (29, 30), which has been considered as a predictor of health and well-being (31).

#### Social isolation

We used two different questions assessing participants’ quantity and quality of social interactions. The first question was: “How many social interactions, on average, did you have in the past week?.” Social interactions could be face-to-face, *via* telephone or online. The second question was: “On average, how satisfied are you with the social interactions of the past week?.” For both questions participants were asked to report a number from 1 (not at all) to 100 (very). Further, we used the Lubben Social Network Scale (LSNS-6) (32), a six-item self-report questionnaire assessing perceived social support received by friends and family. Lastly, the 20-item UCLA scale was employed to measure participants’ feeling of loneliness (33).

#### Food diary *via* FoodApp

Participants could input using a smartphone FoodApp when they had a meal (date and time), the type of meal (e.g., lunch, dinner snack), food item, and quantity (in grams or milliliters). They were asked to complete the daily food diary for 7 consecutive days (34). The output allowed us to compute two main variables: caloric content and information on micro- and macronutrients of the consumed food using the German Federal Food Key data table (Bundeslebensmittelschlüssel; Dehne et al. (35)). We calculated energy intake adjusted values (g/1,000 kcal/day) to account for an individuals’ total energy intake (36). Furthermore, we extracted daily energy derived from each macronutrient. In particular, the daily intake of carbohydrates (g/day) was multiplied by 4 kcal, while fat intake by 9 kcal (22). Lastly, tyrosine and tryptophan to large neutral amino acids (LNAA) ratios were calculated by dividing the quantity of tyrosine and of tryptophan by the sum of the other LNAAs (22, 37, 38).

### Statistical analyses

The analysis plan was preregistered on the public data repository Open Science Framework.^3^ The data was analyzed using R statistical software (R Core Team). Mediation analyses were performed using JASP (version 0.14.1.0). The Shapiro–Wilk test was undertaken to demonstrate that data were normally distributed.

Spearman correlations were performed to test possible associations between each of the variables among social isolation (UCLA, Lubben Scale, self-report measures of quantity, and quality of social interactions), PLEs (CAPE), mental well-being (PSQ-20), and beliefs in conspiracy theories (GCB). Correlations were corrected for multiple comparisons separately for each results section using the Bonferroni method. A mediation analysis was performed to assess if the variable social isolation was mediating the relationship between PLEs and the dependent variable beliefs in conspiracy beliefs. A further mediation analysis was performed using PLEs as a mediator in the relationship between social isolation and conspiracy beliefs. Bootstrapping (1,000 samples) was performed as implemented in the “lavaan” package (39) in JASP.

A Wilcoxon signed-rank test was performed to examine within-group differences in CAPE scores between two different time-points (10 November–23 December 2020 vs. 29 January–8 February 2021). Spearman correlations were performed to test associations between participants’ CAPE scores and GCB scores with their daily food intake ratings.

## Results

An initial sample of 147 participants signed up *via* Prolific. Five participants were excluded from the analyses, as they were not residing in Germany or Austria (see the section “Materials and methods”). Thus, analyses on questionnaire data were performed on the resulting 142 participants (see Table 1 for descriptive statistics). Of those participants, a total of 126 completed their food intake for at least 3 days using the food-diary app. Hence, analysis including food measures was conducted on these 126 participants.

### Psychotic-like experiences and conspiratorial beliefs

**FIGURE 2:**
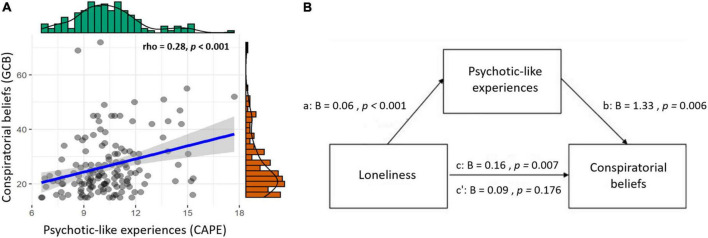
Associations between loneliness, psychotic-like experiences (PLEs) and conspiratorial beliefs. **(A)** Significant positive correlation between conspiratorial beliefs and PLEs (rho = 0.28, *p* < 0.001); **(B)** Mediation model with loneliness as predictor and PLEs as mediator predicting conspiratorial beliefs. Betas are unstandardized, total effect (c), direct path (c’).

We first preregistered to test whether PLEs are associated with conspiratorial beliefs. To do so, we performed a Spearman correlation between the CAPE-total score and the GCB score. Results showed a significant positive correlation between the two questionnaires (rho = 0.28; *p* < 0.001). Thus, the more participants presented PLEs the more they tended to believe in conspiracy theories (see Figure 2A). Further, we assessed which of the three CAPE subscales (positive, negative, and depressive) was correlating with GCB scores. We found that both CAPE-positive (*rho* = 0.45; *p* < 0.001) and negative (*rho* = 0.25; *p* = 0.008) subscales positively correlated with GCB scores, while CAPE-depressive did not (*rho* = 0.15; *p* = 0.231). *P*-values are Bonferroni corrected. These results support a relationship between PLEs (and their positive and negative dimensions) and conspiratorial beliefs.

### Loneliness, psychotic-like experiences, and their influence on conspiratorial beliefs

So far, we showed that PLEs are associated with conspiratorial beliefs. Next, we aimed to investigate whether perceived social isolation in a period of social restrictions is associated with conspiratorial beliefs and PLEs.

Hence, we performed different Spearman correlations between the total scores of questionnaires assessing loneliness (UCLA), social support (LSNS), and self-report measures of quantity and quality of social interactions with GCB scores. Results showed a significant positive correlation only between UCLA and GCB (*rho* = 0.28; *p* = 0.004). No other significant correlations emerged (all *p*’s > 0.38) see Supplementary Table 1. Similarly, UCLA was the only measure of social isolation among others also correlating with CAPE-total scores (*rho* = 0.37; *p* < 0.001) (all *p*’s > 0.69) see Supplementary Table 1.

Overall, these results suggest positive associations between loneliness (regardless of the amount of social interaction and/or social support the participants received), PLEs, and conspiratorial beliefs. These results persisted even when controlling for demographics such as gender and the level of education. More in details, GCB and CAPE total score are still significantly positively associated (rho = 0.272; *p* = 0.002), as well as UCLA and GCB (rho = 0.255; *p* < 0.003), and UCLA and CAPE total score (rho = 0.341; *p* < 0.001). To better understand how loneliness and PLEs relate to conspiratorial beliefs, we preregistered to explore different mediation analyses (see the section “Materials and methods”). More in detail, we first tested whether UCLA could mediate (mediating variable) the relationship between CAPE (predictor variable) and GCB (dependent variable). Results from this analysis showed that UCLA did not mediate the effect of CAPE on GCB [bootstrapped indirect effect (a*b) *B* = 0.26, SE = 0.20, *Z* = 1.31, *p* = 0.189]. The result of this mediation model persisted even when controlling for demographic variables such as gender and level of education [bootstrapped indirect effect (a*b) *B* = 0.24, SE = 0.20, *Z* = 1.21, *p* = 0.23]. Next, a second mediation analysis was performed using CAPE as a mediator in the relationship between UCLA (predictor variable) and GCB (dependent variable). Results showed that CAPE fully mediated the effect of UCLA on GCB [bootstrapped indirect effect (a*b) *B* = 0.08, SE = 0.03, *Z* = 2.57, *p* = 0.012; see Figure 2B], meaning that the more participants felt lonely, the more they believed in conspiracy theories, but this was dependent on their propensity to have PLEs. This mediation model persisted when controlling for demographics such as gender and level of education [bootstrapped indirect effect (a*b) *B* = 0.08, SE = 0.03, *Z* = 2.58, *p* = 0.010].

### Perceived stress is not related to conspiratorial beliefs

Next, we examined if also another factor related to health and well-being such as perceived-stress (PSQ-20) is associated with different levels of conspiratorial beliefs. Correlations were performed using both PSQ-20 total scores and PSQ-20 subscales scores (worries, joy, tension, and demands). A Spearman correlation between PSQ-20 and GCB scores did not reveal a significant result (*rho* = 0.15; *p* = 0.380). No significant results emerged also between PSQ-20 subscales and GCB (all *p*’s > 0.22) see Supplementary Table 2. These results suggest that conspiratorial beliefs are specifically associated with PLEs and loneliness but not with a more general subjective well-being or distress.

### Exploratory analyses

**FIGURE 3:**
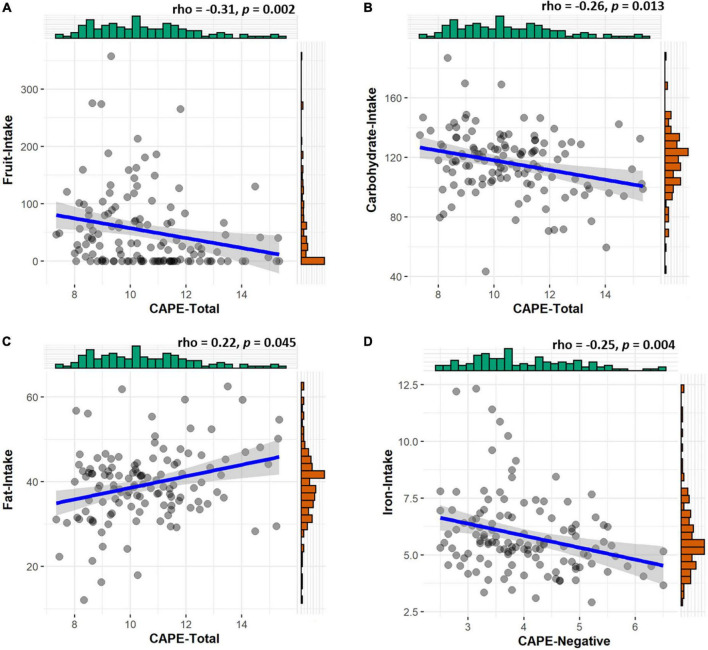
Associations between psychotic-like experiences (PLEs) and food intake. PLEs negatively correlate with **(A)** fruit (rho = −0.31, *p* = 0.002), **(B)** carbohydrate (rho = −0.26, *p* = 0.013), and **(D)** iron (rho = −0.25, *p* = 0.004) intakes. They also positively correlate with **(C)** fat intake (rho = 0.22, *p* = 0.045).

Since a large body of literature has shown an association between dietary intake and the severity of psychotic symptoms in patients with schizophrenia (40), we preregistered to explore whether diet also relates to PLEs in healthy individuals. In particular, we focus on large amino acids such as tyrosine, tryptophan, as well as on iron intake levels since they all have been reported to be involved in the dopaminergic and serotoninergic transmission in the brain and in the pathophysiology of psychosis (12, 37, 40). Based on previous studies on schizophrenia (40), we also examined whether food intake indexed by certain nutrient compositions (e.g., carbs, fat, fruit, and vegetables) is associated with CAPE. More in details, high total intake of fruit and vegetables has been associated with better mental health (22, 41, 42). In line with this evidence, several studies reported a negative association between dietary intake of fruits and vegetables and the presence of psychosis (40, 41, 43–45). Furthermore, studies on stress in animals and humans have found that stress can modify the diet by preferring high-fat and high-carb foods (46, 47). Importantly, psychological stress is often comorbid with schizophrenia (48, 49) and correlates positively with PLEs (50). Regarding the dopaminergic precursor tyrosine and the serotoninergic precursor tryptophan, studies have reported their crucial role in motivation and mood, respectively. For example, it has been shown that acute tyrosine and tryptophan depletions can reduce motivation for reward and lower mood (22, 37, 51, 52). Simultaneously, dopamine and serotonin play an important role in psychosis (53, 54). Based on these results, dietary tyrosine and tryptophan intake levels may be associated with different levels of PLEs. Spearman correlations showed that CAPE scores were not associated with estimated Tyrosine/LNAA, Phenylalanine/LNAA, and Tryptophan/LNAA intakes (all *p*’s > 0.73) see Supplementary Table 3. Interestingly, CAPE scores negatively correlated with fruit (*rho* = −0.31; *p* = 0.002) and carbohydrate (*rho* = −0.26; *p* = 0.013) intakes, and positively correlated with fat intake (*rho* = 0.22; *p* = 0.045) see Figure 3. No significant associations were found between CAPE scores and vegetable intake (*rho* = 0.00; *p* = 1) see Supplementary Table 4. Lastly, CAPE scores negatively correlated with iron intake (*rho* = −0.25; *p* = 0.004) (see Figure 3). Further decomposing this correlation, by performing separate correlations between the different CAPE subscales (positive, negative, and depressive) and iron intake, showed that lower CAPE-negative symptoms were associated with reduced iron intake (*rho* = −0.28; *p* = 0.005) (see Figure 3D). No significant correlations emerged with the CAPE-positive (*rho* = −0.17; *p* = 0.171) and the CAPE-depressive (*rho* = −0.14; *p* = 0.374) subscales (see Supplementary Table 5). These results suggest that the negative dimension of PLEs in healthy individuals is associated with lower iron intake. Strikingly, these results are in line with studies on patients with chronic psychotic disorders (12, 40). Lastly, no significant associations emerged between GCB and food intake (all *p*’s > 0.38) see Supplementary Tables 6–8.

Note that food intake measures and CAPE scores used in these analyses were collected during time-point one (10 November–23 December 2020), while all the other questionnaire measures were collected during time-point two (29 January–8 February 2021) (see Figure 1). Since the same participants were asked to fill out the CAPE questionnaire during both time-points, a Wilcoxon signed-rank test was performed to assess whether their levels of PLEs changed over time. Results showed no differences in CAPE scores between the two time-points (time-point one *M* = 10.51, *SD* = ± 1.84; timepoint two *M* = 10.40, *SD* = ± 0.91; *V _Wilcoxon_* = 4490, *p* = 0.306). Since we did not exclude participants outside of the normal BMI range, we tested through correlations and mediation analyses whether this variability may (or may not) impact our results. These analyses seem to suggest that the variability of participants’ BMI did not impact our results (see Supplementary materials).

## Discussion

This preregistered study investigated whether PLEs are associated with conspiratorial beliefs during the lockdown in a global health crisis. As an emerging field of research (3), only a few studies have investigated the possible relationship between conspiratorial beliefs and PLEs (7, 55, 56). We were also interested in other pandemic-related factors such as social isolation as a possible contributor to conspiratorial beliefs since social restriction measures were so prominent during lockdowns. Therefore, we assessed whether both PLEs and social isolation can predict conspiratorial beliefs and, if so, which of these variables can mediate the association with conspiratorial beliefs. Furthermore, based on previous studies on schizophrenia, we explored whether the diet is associated with PLEs and conspiratorial beliefs.

We hypothesized that PLEs are associated with conspiratorial beliefs. Similarly, we hypothesized that also other pandemic-related factors such as social isolation and mental well-being are associated with conspiratorial beliefs. Lastly, we hypothesized that PLEs, social isolation and mental well-being could all be associated with each other and predict conspiratorial beliefs. If so, we tested through different mediation analyses whether one of these variables can mediate the contribution of the other in predicting the endorsement of conspiracy beliefs.

In line with our hypothesis, results show that PLEs are positively associated with conspiratorial beliefs, meaning that the higher the participants’ levels of PLEs the more they reported to endorse conspiratorial beliefs. This result provides an extension of previous research, showing an association between a subcomponent of PLEs such as paranoia and the endorsement of conspiracy theories (2, 7). It has been argued that similar to individuals with high levels of PLEs, those supporting conspiratorial beliefs tend to collect less information to make decisions (jumping to conclusion bias). Therefore, both PLEs and conspiratorial thinking may have in common a more intuitive thinking style (2). In line with this observation, studies have found negative associations between analytic thinking and the endorsement of conspiratorial beliefs (57, 58). Interestingly, we found that not only the positive dimension of PLEs (e.g., paranoia) but also its negative dimension (e.g., avolition or lack of motivation) is associated with conspiratorial beliefs. This association was not found with the depression dimension (e.g., affective component) of PLEs. Overall, these findings suggest that not all subdimensions of PLEs are associated with conspiratorial beliefs and that both the positive (possibly through cognitive processes such as the jump to conclusion bias) and negative (reduced motivation) dimensions of PLEs may contribute to believing in conspiracy theories. In line with these findings, a study by Ståhl and colleagues (59) proposed that skepticism toward conspiratorial beliefs requires sufficient cognitive and motivational abilities, which are both altered in individuals with high levels of PLEs (9, 59, 60).

Another important result that emerged from our study is the role of loneliness in the endorsement of conspiracy theories. During the COVID-19-pandemic, social distancing restrictions led some people to experience greater social isolation and mental health illnesses (61). It is currently unknown what role social isolation plays in the dynamic between PLEs and conspiratorial beliefs in the context of the pandemic. Interestingly, loneliness positively predicted both PLEs and conspiratorial beliefs. However, this association was not found with other measures of social isolation such as social support and measures of quantity and quality of social interactions. Therefore, although studies showed that these measures of social isolation are highly correlated (62), our results suggest that only the subjective feeling of a lack of satisfactory interpersonal relationships (and not the objective amount of social support) is related to PLEs and conspiratorial beliefs during difficult times such as the COVID-19 pandemic. Similar associations between loneliness, PLEs, and conspiratorial beliefs were found in a previous study (7). The authors argued that the increased feelings of loneliness may have led people more susceptible to hear voices or perceive humanlike agency also in non-human stimuli (63), eventually influencing their association with conspiratorial beliefs (7). Our mediation analysis could confirm this hypothesis by showing that the proneness to show PLEs fully mediated the relationship between loneliness and conspiratorial beliefs. That is, the experience of loneliness during the COVID-19 pandemic enhances the proneness to experience psychotic events that increases the endorsement of conspiracy theories.

Besides social isolation and PLEs, the lockdown also resulted in diet changes (64). Research shows that a healthy diet helps to protect mental health (65). However, no studies have investigated the link between diet, PLEs and conspiratorial beliefs during challenging, and stressful times. We explored whether food intake, and in particular iron intake levels, may be associated with PLEs and conspiratorial beliefs. We found that food intake was not associated with conspiratorial beliefs. However, in line with studies on patients with schizophrenia, we found a significant association between food intake and PLEs levels. More in detail, PLEs were negatively associated with fruit, carbohydrate, and iron intakes, and positively with fat intake. In line with our findings, some studies have reported improved symptoms or decreased incidence/risk of schizophrenia with higher dietary fruit intake (40), possibly due to the antioxidant and anti-inflammatory activity of a diet rich in fruits (66). Differently, the association between psychosis and total dietary carbohydrates and fat intakes is unclear, with some studies showing a positive association (67, 68), while others a negative association or no association (40). In addition, it has been reported that altered iron homeostasis is implicated in neuropsychiatric disorders (69). In particular, iron reductions can result in changes in dopamine neurotransmission and altered neurodevelopment (70). Indeed, prospective studies have shown a significant relationship between maternal iron deficiency and the risk of schizophrenia in offspring (71, 72). Interestingly, first-episode schizophrenia individuals with high levels of negative symptoms showed lower levels of blood iron compared to healthy controls (12). Similarly, a magnetic resonance imaging (MRI) study found a decreased iron concentration in gray matter nuclei including the bilateral substantia nigra in first-episode schizophrenia individuals compared to healthy controls (14). In line with these results, we found that higher levels of PLEs (in particular, the negative domain of PLEs) are associated with a reduced average daily iron intake. Overall, our findings suggest a possible link between reduced iron intake and PLEs, possibly influencing dopaminergic neurotransmission in the brain and therefore accounting for these subclinical symptoms in the general population.

Some limitations of the current study should be addressed. First, loneliness, conspiratorial beliefs and food intake were only measured once, therefore we cannot assess within-person changes over time. Second, conspiratorial beliefs and food intake were not time-locked. Third, our findings are correlational, and we cannot make causal arguments. Fourth, our measures were based on self-reports, which may have lower reliability and validity.

## Conclusion

In conclusion, loneliness predicted the endorsement of conspiracy theories during the COVID-19 lockdown. Strikingly, the proneness to experience subclinical psychotic symptoms played an underlying mediating role. In addition, these subclinical symptoms were associated with lower fruit, carbohydrate, and iron intakes, as well as with higher fat intake. Our results contribute to the study of beliefs in conspiracy theory and add insights into how they can affect individuals’ mental health and relationships. Moreover, these results open the avenue for potential novel intervention strategies to manage and optimize food intake in individuals with PLEs. In future research, experimental designs should be used to test the possible causal effects shown in this study.

## Data availability statement

The raw data supporting the conclusions of this article will be made available by the authors, without undue reservation.

## Ethics statement

The studies involving human participants were reviewed and approved by The Humbold Ethics Committee approved the study. The patients/participants provided their written informed consent to participate in this study.

## Author contributions

DT and SP: conceptualization and project administration. DT: investigation, writing – original draft preparation, and visualization. DT, AL, and SP: methodology. DT, A-KM, and AL: formal analysis. A-KM, AL, DT, and SP: writing – review and editing. SP: supervision and funding acquisition. All authors have read and agreed to the published version of the manuscript.

## Footnotes

1
https://osf.io/nqhjf

2
https://www.prolific.co/

3
https://osf.io/y36q9
