# Supplementary material for: Psychotic-like experiences in the lonely predict conspiratorial beliefs and are associated with the diet during COVID-19
Source: Front Nutr. 2022 Oct 28;9:1006043. doi: 10.3389/fnut.2022.1006043 (PMC9650276; doi:10.3389/fnut.2022.1006043)

***Supplementary Materials***

| **Variable** | **Variable** | **Rho** | ***p*** |
| --- | --- | --- | --- |
| GCB  CAPE-total | Quantity Social Interactions | -0.04 | > 0.999 |
|  | Quality Social interactions | -0.14 | 0.379 |
|  | UCLA | 0.28 | **0.004** |
|  | LSNS | -0.05 | > 0.999 |
|  | Quantity Social Interactions | -0.03 | > 0.999 |
|  | Quality Social interactions | -0.19 | 0.094 |
|  | UCLA | 0.37 | **< 0.001** |
|  | LSNS | -0.12 | 0.687 |

**Supplementary Table 1**. Spearman correlations between conspiratorial beliefs and psychotic-like experiences with measures of social isolation.

| **Variable** | **Variable** | **Rho** | ***p*** |
| --- | --- | --- | --- |
| GCB | PSQ-20 total | 0.15 | 0.380 |
|  | PSQ-20 worries | 0.14 | 0.478 |
|  | PSQ-20 joy | -0.17 | 0.224 |
|  | PSQ-20 tension | 0.14 | 0.488 |
|  | PSQ-20 demands | 0.08 | > 0.999 |

**Supplementary Table 2.** Correlations between conspiratorial beliefs and perceived stress.

| **Variable** | **Variable** | **Rho** | ***p*** |
| --- | --- | --- | --- |
| CAPE-total | Tyrosine/LNAA | -0.10 | 0.729 |
|  | Phenylalanine/LNAA | 0.00 | > 0.999 |
|  | Tryptophan/LNAA | 0.08 | > 0.999 |

**Supplementary Table 3.** Correlations between psychotic-like experiences and estimated dietary amino acids intake.

| **Variable** | **Variable** | **Rho** | ***p*** |
| --- | --- | --- | --- |
| CAPE-total | Fruit | -0.31 | **0.002** |
|  | Vegetable | 0.00 | > 0.999 |
|  | Carbohydrate | -0.26 | **0.013** |
|  | Fat | 0.22 | **0.045** |

**Supplementary Table 4.** Correlations between psychotic-like experiences and estimated dietary fruit, vegetables, carbohydrate, and fat intakes.

| **Variable** | **Variable** | **Rho** | ***p*** |
| --- | --- | --- | --- |
| CAPE-total  CAPE-positive  CAPE-negative | Iron | -0.25 | **0.004** |
|  | Iron | -0.17 | 0.171 |
|  | Iron | -0.28 | **0.005** |
| CAPE-depressive | Iron | -0.14 | 0.374 |

**Supplementary Table 5.** Correlations between psychotic-like experiences and estimated dietary iron intake

| **Variable** | **Variable** | **Rho** | ***p*** |
| --- | --- | --- | --- |
| GCB | Tyrosine/LNAA | -0.12 | 0.556 |
|  | Phenylalanine/LNAA | -0.19 | 0.095 |
|  | Tryptophan/LNAA | 0.07 | > 0.999 |

**Supplementary Table 6.** Correlations between conspiratorial beliefs and estimated dietary amino acids intake

| **Variable** | **Variable** | **Rho** | ***p*** |
| --- | --- | --- | --- |
| GCB | Fruit | -0.05 | > 0.999 |
|  | Vegetable | -0.05 | > 0.999 |
|  | Carbohydrate | -0.02 | > 0.999 |
|  | Fat | 0.04 | > 0.999 |

**Supplementary Table 7.** Correlations between conspiratorial beliefs and estimated dietary fruit, vegetables, carbohydrate, and fat intakes

| **Variable** | **Variable** | **Rho** | ***p*** |
| --- | --- | --- | --- |
| GCB | Iron | -0.09 | 0.317 |

**Supplementary Table 8.** Correlation between conspiratorial beliefs and estimated dietary iron intake

**Body Mass index variability**

Since we did not exclude participants outside of the normal BMI range, we tested through correlations and mediation analyses whether this variability may (or may not) impact our results. Most of the participants was normal weight (93 participants, 65.96% of the whole sample), while 9 participants (6.38%) were underweight (BMI <18.5), 28 participants (19.86%) were overweight (BMI ranging from 25 to 29.9), and 11 participants (7.80%) were obese (BMI ≥ 30). To test for possible associations between participants’ BMI and our main variables of interest, we performed different Spearman correlations. Results showed that BMI was not associated with (all p’s > 0.37) psychotic-like experiences (including CAPE total score and its subscales), measures of social isolation (quantity and quality of social interactions, UCLA, and Lubben scale), mental well-being (PSQ-20), and conspiratorial beliefs (GCB scale).
We also included BMI as a nuisance covariate in our main mediation analysis to test whether our results persisted even when controlling for this variable. Results showed also in this case that CAPE fully mediated the effect of UCLA on GCB (bootstrapped indirect effect (a*b) B = 0.076, SE = 0.030, Z = 2.508, p = 0.012). Overall, these analyses seem to suggest that the variability of participants’ BMI did not impact our results.

**Methods**

***Food diary via FoodApp***

Estimated tyrosine and tryptophan to LNAA ratios were computed by dividing the quantity of tyrosine and of tryptophan by the sum of the other LNAAs (equations 1a, 1b, 2).


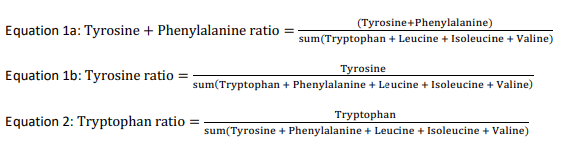

Supplement: Supplementary file 1 [file Data_Sheet_1.docx]
